# 18 beta-glycyrrhetinic acid ameliorates the cognitive functions and decreases the recurrence rate of pituitary adenomas patients

**DOI:** 10.17179/excli2018-1330

**Published:** 2018-07-27

**Authors:** Xianxiang Wang, Yiquan Zhang, Jin Xiao, Ke Zhang, Qingxin Li, Hongwei Chen, Fei Liu

**Affiliations:** 1 Department of Neurosurgery, the First Affiliated Hospital of Anhui Medical University, Hefei 230022, China

**Keywords:** pituitary adenoma, cognitive function, 18 beta-glycyrrhetinic acid (GA), recurrence rate

## Abstract

Pituitary adenomas, the most common tumors of all intracranial neoplasms, may cause either symptoms of mass effect or symptoms of hormone abnormal production. Besides physical damages, patients with pituitary adenomas always suffer from cognitive impairments, mainly in memory and executive functions. 18 beta-glycyrrhetinic acid (GA) has been found to exhibit anti-tumor effects in rat pituitary adenoma-derived cells. The aim of this paper was to investigate the effect of GA in postoperative clinical application. In this study, we recruited 647 patients with pituitary adenoma and 135 patients who dropped out were excluded from analysis. Thus, altogether 512 patients with pituitary adenoma completed the study, of whom 268 were treated with GA, and 244 were treated with placebo. Cognitive assessments, eyesight, tumor size, and hormone secretion levels were examined before and after surgery in both groups. All patients underwent surgeries by single-nostril transsphenoidal approach for their first-time medical treatment. Hormone secretion levels were measured by blood samples and Magnetic resonance imaging (MRI) was used for examining the status of tumor excision. Compared with placebo group, the scores in orientation, language (expression), memory (recall), practice, abstract thinking, and MiniMental State Examination (MMSE) were significantly improved (p < 0.05) in GA treated group after one month of surgery. After six months, there was still a significant increase in abstract thinking scores. Moreover, GA did not impact the overall survival percentage of patients enrolled during our five-year follow-up, but significantly reduced the recurrence rate than that of the placebo group. GA significantly improved the cognitive functions at the early stage after surgery and had a long-termed efficacy on abstract thinking. Notably, GA inhibited the five-year recurrence rate of the recruited pituitary adenomas patients.

## Introduction

Pituitary adenomas are tumors that occur in the pituitary gland and account for the largest portion of all intracranial neoplasms with an overall prevalence of approximately 10 % to 25 % (Ezzat et al., 2004[8]). Depending on biological functioning, pituitary adenomas are commonly divided into three categories: benign adenoma, invasive adenoma, and carcinomas (Zhang, 1990[32]). The majority of adenomas are benign, while approximately 35 % are invasive adenoma and just 0.1 % to 0.2 are carcinomas (Daly et al., 2006[5]). However, only a minority of pituitary adenomas are symptomatic. The most characteristic-presenting symptoms of pituitary adenomas include visual deficits and abnormal pituitary hormone secretion (Levy et al., 2005[20]). Moreover, pituitary adenomas have been found to be associated with pituitary disorders and various psychiatric manifestations such as emotional instability, depression, apathy, anxiety, easy hostility and irritability (Donoho et al., 2017[7]). The primary treatments of pituitary adenomas involve adjustment of hormone secretion and resection of the tumor (Wang et al., 2017[28]). Reducing hormone hypersecretion and correcting hormone deficiencies may improve clinical symptoms and decreasing tumor size by surgery may relieve the mass effect of pituitary adenomas (Pineda et al., 1982[22]). Specific treatment plans for pituitary adenomas patients are made depending on the specific type of tumor, and administered with a comprehensive therapy approach that include neurosurgery and endocrinology (Wang, 1988[27]). Transsphenoidal adenectomy is a common strategy for the excision of pituitary tumors, which aims to remove the tumors without affecting the function of optic nerves and the brain (Pituitary Disorders/Neuroendocrinology, 2016[23]). However, in numerous patients, surgery has been found to inevitably damage the function of the brain and impair the cognitive functions (Karki et al., 2017[16]). Moreover, long-term use of endocrine drugs might cause various adverse effects such as fatigue, nausea, and vomiting (Zhang et al., 2017[33]). In addition, incomplete resection and recurrence are still obstacles to the treatment of pituitary tumors (Vonor et al., 2017[25]). Therefore, finding proper postoperative adjuvant drug therapy is in special need.

Recent studies have found that 18 beta-glycyrrhetinic acid (GA), a natural compound extracted from liquorice, inhibits renal 11beta-hydroxysteroid dehydrogenase type 2 (11beta-HSD2) and exhibits significant anti-tumor effects on pituitary adenoma (Wang et al., 2014[26]). GA induced apoptosis in pituitary adenoma cells by activating mitochondria-mediated reactive oxygen species (ROS)/mitogen-activated protein kinase pathways (Wang et al., 2014[26]). In particular, GA activated c-Jun N-terminal kinase (JNK), calcium/calmodulin-dependent protein kinase II (CaMKII), and P38 (Wang et al., 2014[26]). These findings provide experimental evidence that GA may serve as a novel chemotherapeutic agent for treating pituitary adenoma.

In the presented study, we recruited 647 pituitary adenoma patients who underwent surgery to further investigate the effects of GA treatment. Patients' cognitive functions were tested when treated with GA or placebo one month and six months after surgery. This paper aimed to explore the role of GA in postoperative clinical recovery of pituitary adenoma patients.

## Materials and Methods

### Patients

In this study, we recruited 647 patients with pituitary adenoma, of whom 339 were treated with GA, and 308 were treated with placebo. Altogether 512 patients completed the study, of whom 268 were treated with GA, and 244 were treated with placebo. 135 patients who dropped out were excluded from analysis, the dropout rates of the GA and the placebo group were 20.9 % and 20.8 %. Orally administered GA was well tolerated in all patients who concluded the trial. Dropout reasons during the study were: Death (8 patients on placebo and 10 patients on GA), withdrawal of consent (14 patients on placebo and 18 patients on GA), loss of contact (30 patients on placebo and 36 patients on GA), improper usage of the treatments (12 patients on placebo and 7 patients on GA).

The ethics committee of the First Affiliated Hospital of Anhui Medical University has proved all studies in this paper. All patients were selected based on following criteria. Patients aged between 18 and 60 years who were diagnosed as pituitary adenoma by pathological examination. Notably, patients selected in this paper underwent their first-time medical visit and treatment. Surgeries were performed using the single-nostrilendonasal transsphenoid-sella approach. The enrolled patients underwent successful surgery procedure. More than 60 % of tumor tissue was excised which was tested by head Magnetic resonance imaging (MRI) examination and no severe complications appeared. In addition, the surrounding tissues had no compression after 3 months of surgery (no pressure on optical nerve as previously described). 

Patients with the following symptoms need to be excluded: (1) patients with pulmonary diseases, cardio-cerebral diseases (including epilepsy, cerebral infarction, and intracerebral hemorrhage), or other complications that severely impaired cognitive functions; (2) patients with severe complications after surgery, such as cerebral hemorrhage, infection, epilepsy, hyperpyrexia, coma, leaking of cerebrospinal fluid and reoperation on bleeding in surgical cavity; (3) patients who could not write and could not see clearly on books; (4) patients with aphasia or hearing loss. 

After permission, patients' clinical data were collected, including age, sex, educational level, visual acuity (ophthalmologic examination), tumor size (in mm, based on MRI report), disease progression (occurrence of signs and symptoms in the patient's complaint). Hormonal secretion tests including cortisol, growth hormone, prolactin (PRL), adrenocorticotropic hormone (ACTH), thyroxine (T3andT4), thyrotropin (TSH), estradiol, testosterone, follicle stimulating hormone (FSH), and luteinizing hormone (LH) were examined by using patients' blood samples collected in the morning after an overnight fastening. On the same day, cognitive assessment was performed to obtain a preoperative cognitive function score. All of the above checks are necessities without additional burdens. All patients underwent standard transsphenoidal surgery under general anesthesia through tracheal intubation, using microscopic or endoscopic examination.

All patients were randomly assigned to either GA (500 mg orally once daily; MAFCO) or placebo (500 mg dextrose orally once daily) treated group without prior knowledge to rule out potential bias.

### Cognition evaluation

The hormone test was performed on the same day with the consent of the patient and their family member. All patients were examined in the same room by the same professionally trained doctor and accompanied by a family member. During the examination, the patient should not pose any gesture or speak. The examinations were taken in the afternoon and lasted for about half an hour. The examinations were immediately terminated if there were any discomforts to patients and the patients were subsequently excluded. Language (comprehension and expression), orientation, attention, abstract thinking, calculation, memory (recent, remote and recall memory), practice, and perceptions were tested using Cambridge Cognitive Examination-Chinese version (CAMCOG-C) and MiniMental State Examination (MMSE) was used in this study. With consent, patients with a score of equal to or less than 90 in CAMCOG-C underwent further examinations by using the head doppler tissue imaging (DTI).

### Data analysis

All values are shown as x ± SEM for normal distribution and medium value (M, QU − QL) for non-normal distribution. QU is the 3rd quantile and QL is the 2nd quantile. Categorical and binary variables are presented as frequencies (percentages). Comparison between groups was performed using Student's t test (unpaired). Proportions were compared using the Fisher exact test. Differences with p<0.05 were considered significant. 

## Results

### Clinical features and functional response of recruited patients

In this study, we recruited 647 patients with pituitary adenoma and 135 patients who dropped out were excluded from analysis. Thus, altogether 512 patients with pituitary adenoma completed the study, of whom 268 were treated with GA, and 244 were treated with placebo (Table 1(Tab. 1)). Reasons for dropout during the study are listed in the methods section. All patients underwent first-time medical surgery after diagnosed and all treatments were suitable for patients. Both GA and placebo treated group shared almost equivalent age distribution, gender distribution and tumor size. In addition, the percentages of functioning and non-functioning pituitary adenoma patients are similar in both groups. Table 1(Tab. 1) showed that both GA treated group and placebo treated group possess unanimous clinical features. We also examined the functional response of patients before surgery, including orientation, language (comprehension, expression), memory (remote, recent, recall), attention, practice, abstract thinking, calculation, perception and MMSE. We found that all cognitive function test scores of patients in GA treated group shared no significant difference with that in the control placebo group (Table 2(Tab. 2)). 

### Cognitive function changes after surgery

We first evaluated the cognitive function changes one month after surgery. As listed in Table 3(Tab. 3), compared with the placebo group, the scores in orientation, language (expression), memory (recall), practice, abstract thinking, and MMSE were significantly higher (p < 0.05) in the GA treated group. When six months after surgery, only the abstract thinking scores exhibited significant improvement in GA treated group, whereas other cognitive function scores showed no notable differences between GA and placebo treated group six months after surgery, indicating that the efficacy of GA is considerable only at the initial stage after surgery. 

### Survival rate and recurrence percentage of recruited patients 

**Figure 1:**
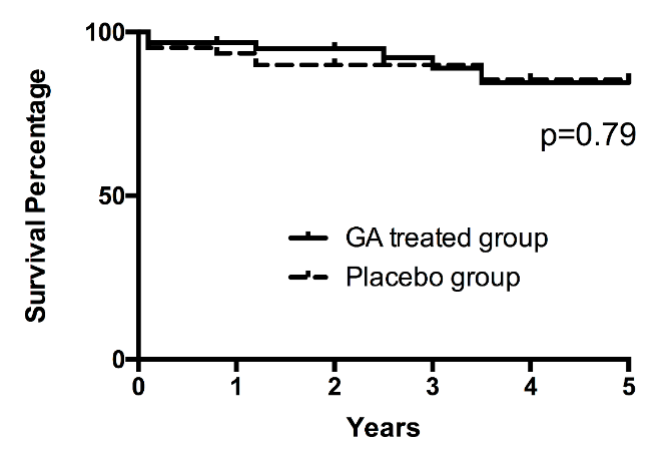
Overall survival rates for patients treated with GA or placebo. Overall survival rate after surgery. There was no significant difference on cumulative survival rate between patients treated with GA and those treated with placebo (p = 0.79).

**Figure 2:**
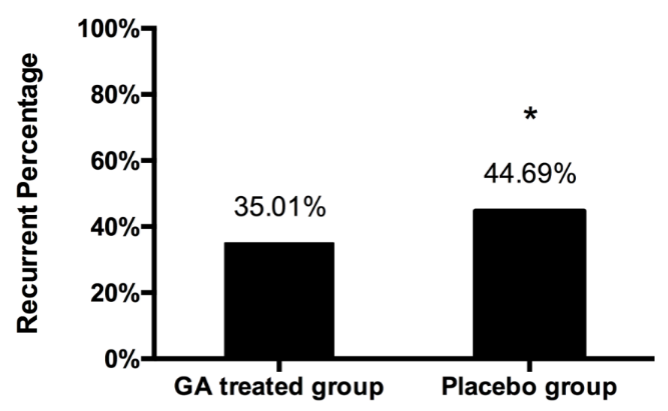
Recurrent percentage after surgery in patients with pituitary adenoma Graph showing percentage of recurrent 5 years after surgery. There was a significant decrease in the recurrence rate in GA treated group than those in placebo group. Proportions were compared using the Fisher exact test. * represents p value <0.005.

As shown in Figure 1(Fig. 1), during our five-year follow-up, the survival rates showed no significant differences between the GA and placebo treated groups (P = 0.79). Since the recurrence rate is generally higher after 5 years of surgery, we compared the differences in recurrence rates between the two groups within 5 years after surgery, and found that the recurrence rate of the GA treated group was significantly lower than that of the placebo group (Figure 2(Fig. 2)). These results indicated that GA might possess potential clinical value on inhibiting the recurrence rate of pituitary adenoma. 

## Discussion

Pituitary adenomas, the most common tumors of all intracranial neoplasms, may cause either symptoms of mass effect or symptoms of hormone abnormal production (Molitch, 2017[21]). Besides physical damages, patients with pituitary adenomas always suffer from cognitive impairments, mainly in memory and executive functions (Kalinin et al., 2017[15]). Surgery has always been considered the best way to treat pituitary adenomas, whereas few studies have been focused on the postoperative treatment of patients (Hendrix et al., 2017[12]). Recent studies have shown that surgeries may inevitably damage the cognitive functions (mainly in executive functions and memory) in patients (Fu et al., 2017[10]). Surgery should not exacerbate the cognitive impairment of patients in theory, so more exhaustive researches are needed to investigate the impact of surgery on cognitive function (Iglesias et al., 2017[14]). Therefore, it is necessary to monitor the changes and to improve the impairment of cognitive function after surgery. In this paper, we recruited patients with pituitary adenomas and tested their cognitive functions when treated with GA or placebo before and after surgery. We aim to investigate the efficacy of GA in postoperative clinical application.

GA and its precursor glycyrrhizin (GN) abundantly exist in the Licorice roots and possess various therapeutic properties (Greaves, 1990[11]). Recent studies have found that GA inhibits 12-O-tetradecanoyl phorbol-13-acetate-induced cutaneous oxidative stress and tumor progression in mice (Agarwal et al., 2005[1]). DMBA/TPA-induced skin tumor formation could also be suppressed by GA (Agarwal et al., 1991[2]). In addition, GA played a protective role in hepatocellular carcinoma development by reducing immunosuppression mediated by hepatic stellate cells in the tumor microenvironment (Kuang et al., 2013[18]). While GA has been shown to exhibit anti-viral and anti-inflammatory effects, it also displays cytotoxic effects on human hepatocellular carcinoma, breast cancer (Wang et al., 2015[29]) and ovarian cancer (Yang et al., 2012[31]). It has been shown that GA induced apoptosis in various cancer cells, including promyelotic leukemia HL-60 cells, human stomach cancer cells, and hepatoma cells (Hibasami et al., 2006[13]). Our study is based on the facts that GA exhibited potent cytotoxicity and induced apoptosis by activating ROS/MAPKs-mediated pathway in pituitary adenoma cells (Wang et al., 2014[26]). These findings suggest that GA may serve as a novel chemotherapeutic potential for treating pituitary adenoma and deserves further clinical research. Previous studies demonstrated the protection of low-dose GA (50 mg/kg) against TP-induced hepatotoxicity in rats, which may mediate via anti-inflammation, antioxidation, and antiapoptosis (Yang et al., 2017[30]). In addition, the protective effect is dose-related, which is only mediated by low-dose GA (50 mg/kg), but not high-dose GA (100 mg/kg) (Yang et al., 2017[30]). Thus, the doses of GA should be carefully considered in treating patients. A recent study examined the kinetics and dynamics of orally administered GA in humans (Krahenbuhl et al., 1994[17]). Different oral doses of GA (500, 1000, or 1500 mg) were administered to healthy volunteers (Krahenbuhl et al., 1994[17]). Based on the single dose kinetics, the kinetic/dynamic analysis revealed that after multiple doses of 1.5. g GA/day, the 11 beta OHSD might be constantly inhibited, whereas at daily doses of 500 mg or less, such an inhibition might occur only transiently (Krahenbuhl et al., 1994[17]). Besides, various previously published pilot studies have applied the dose of 500 mg in human (Farese et al., 2009[9]; Serra et al., 2002[24]). So, a low-dose 500 mg GA was used in our paper. We found that GA exhibited positive effects on orientation, language (expression), memory (recall), practice, abstract thinking, and MMSE one month after surgery, but lost efficacy after six months only except for abstract thinking. Since when six months after surgery, most of the cognitive function scores were higher compared with one month after surgery, so GA might accelerate the recovery of functions in language (expression), memory (recall), practice, and MMSE. Notably, GA possessed a long-termed effect on abstract thinking. These functions might correlate with the anti-pituitary adenoma effect of GA by inducing apoptosis in remaining tumor tissues.

Although pituitary adenomas are commonly considered as benign intracranial neoplasms, they are frequently found to exhibit invasive abilities (Babbo et al., 2014[3]). In numerous patients, peripheral brain tissues including parasellar and suprasellar regions, bilateral cavernous sinus and sphenoid sinus are invaded by pituitary adenomas (Kwancharoen et al., 2014[19]). In addition, pituitary adenomas could even damage brain tissues and structures such as those in the vomer peripheral region and sellar region (Kwancharoen et al., 2014[19]). Thus, pituitary adenomas may possess biological characteristics of malignant tumors, leading to a reduction in complete resection rate and a higher recurrence rate, even being facilitated with radiotherapy or chemotherapy adjuvant therapy (Ding et al., 2014[6]). It is reported that 10 % to 35 % of pituitary adenomas recurred in 4 to 20 years' intervals (Broder et al., 2014[4]). The initial tumor size, the tumor excision extent and the tumor regrowth rate are considered to be determining factors in pituitary adenoma recurrence (Broder et al., 2014[4]). Therefore, the complete treatment of pituitary adenomas is extremely difficult and is now a major health issue that raises a wide range of research concerns. In this paper, we found that GA could significantly reduce the recurrence rate within 5 years after surgery, suggesting that GA could be used as a novel treatment strategy against recurrent pituitary adenoma, although the detailed functional mechanism of GA remains to be further elucidated.

In conclusion, GA significantly improved various cognitive functions at the early stage after surgery and possessed a long-termed efficacy on abstract thinking. In addition, GA inhibited the five-year recurrence rate of the recruited pituitary adenomas patients. These findings suggest that GA might serve as a novel chemotherapeutic potential for inhibiting the recurrence of pituitary adenoma. 

## Acknowledgements

Not applicable.

## Competing interests

The authors declare that they have no competing interests.

## Informed consent

Informed consent was obtained from all individual participants included in the study.

## Research involving human participants and/or animals

All procedures performed in studies involving human participants were in accordance with the ethical standards of the institutional and/or national research committee and with the 1964 Helsinki declaration and its later amendments or comparable ethical standards.

## Figures and Tables

**Table 1:**
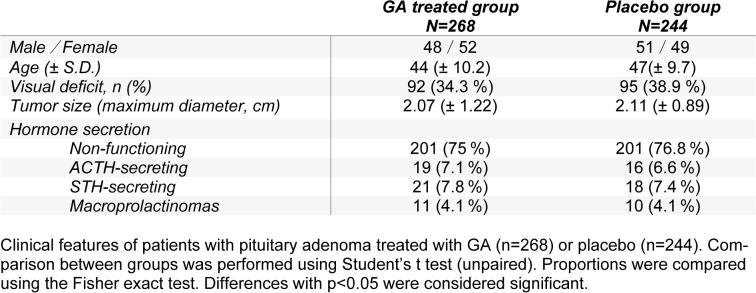
Clinical features of the patients

**Table 2:**
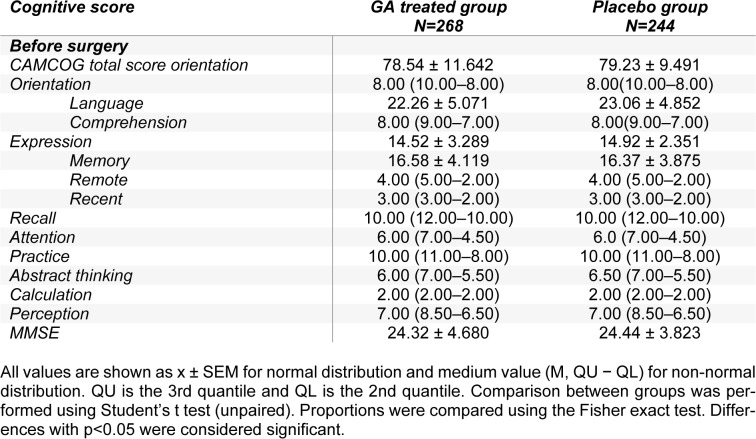
Functional response of patients before surgery

**Table 3:**
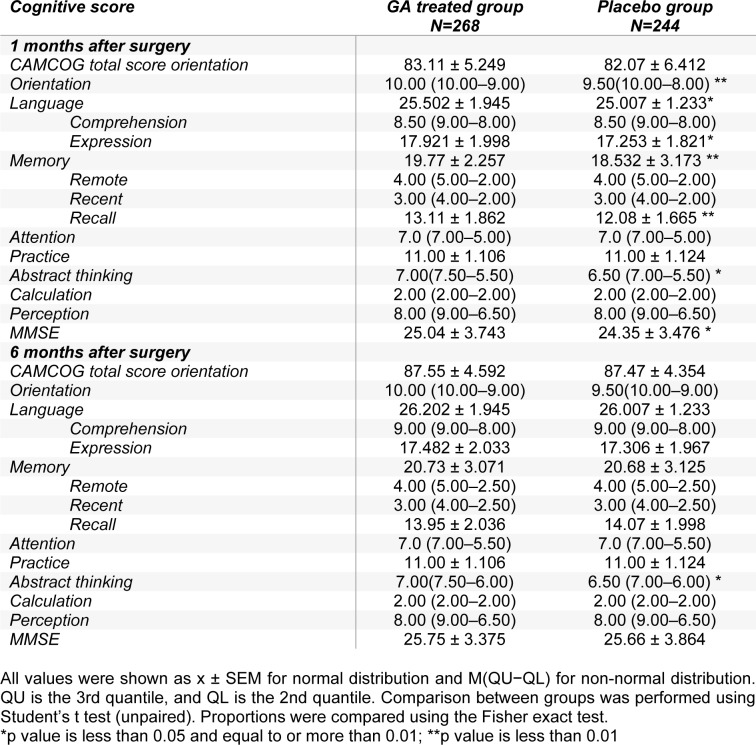
Functional response of patients after surgery
